# Rab27a-Dependent Paracrine Communication Controls Dendritic Spine Formation and Sensory Responses in the Barrel Cortex

**DOI:** 10.3390/cells10030622

**Published:** 2021-03-11

**Authors:** Longbo Zhang, Xiaobing Zhang, Lawrence S. Hsieh, Tiffany V. Lin, Angélique Bordey

**Affiliations:** 1 Departments of Neurosurgery, and Cellular & Molecular Physiology, School of Medicine, Yale University, 333 Cedar Street, New Haven, CT 06520-8082, USA; longbo.zhang@yale.edu (L.Z.); lawrence.hsieh@biogen.com (L.S.H.); tiffany.lin@yale.edu (T.V.L.); 2 Department of Psychology, Florida State University, Tallahassee, FL 32306, USA; xzhang@neuro.fsu.edu

**Keywords:** exosomes, dendritic spine, excitatory synapse, somatosensory cortex, whisker stimulation

## Abstract

Rab27a is an evolutionarily conserved small GTPase that regulates vesicle trafficking, and copy number variants of *RAB27a* are associated with increased risk of autism. However, the function of Rab27a on brain development is unknown. Here, we identified a form of paracrine communication that regulates spine development between distinct populations of developing cortical neurons. In the developing somatosensory cortex of mice, we show that decreasing Rab27a levels in late-born pyramidal neurons destined for layer (L) 2/3 had no cell-autonomous effect on their synaptic integration but increased excitatory synaptic transmission onto L4 neurons that receive somatosensory information. This effect resulted in an increased number of L4 neurons activated by whisker stimulation in juvenile mice. In addition, we found that Rab27a, the level of which decreases as neurons mature, regulates the release of small extracellular vesicles (sEVs) in developing neurons in vitro and decreasing Rab27a levels led to the accumulation of CD63-positive vesicular compartments in L2/3 neurons in vivo. Together, our study reveals that Rab27a-mediated paracrine communication regulates the development of synaptic connectivity, ultimately tuning responses to sensory stimulation, possibly via controlling the release of sEVs.

## 1. Introduction

Rab27 is a member of the small GTPase Rab family, which consists of master regulators of intracellular membrane trafficking in all eukaryotic cells [1,2,3]. A single Rab27 isoform is present in invertebrates and two isoforms, Rab27a and Rab27b, are present in vertebrates [4] and are involved in the exocytic pathway [5,6]. Rab27a was the first identified Rab protein whose dysfunction leads to a human hereditary disease, type 2 Griscelli syndrome [7]. This disease is mostly studied for immunodeficiency and associated hemophagocytic lymphohistocytosis that lead to severe neurological abnormalities [8]. *RAB27A* copy number variants have also been reported in sporadic cases of autism and are thought to play a role in the etiology of simplex autism [9]. In Caenorhabditis (C.) elegans and in squid, Rab27 is expressed in neurons and regulates synaptic transmission [10,11] and knockout of Rab27 in Drosophila leads to an altered sleep phenotype [12], suggesting important functions of Rab27 in the nervous system of these organisms. In mammalian brains, the levels of Rab27a are developmentally regulated [13]. However, a role of Rab27a in the establishment of synaptic circuits in the mammalian brain remains unknown.

Here, we investigated the role of Rab27a on the synaptic integration of cortical pyramidal neurons in mice. Given the high expression of RAB27A in the developing cortex [14], we used in utero electroporation to knockdown Rab27a expression in late-born pyramidal neurons that populate layer (L) 2/3 of the somatosensory cortex. We found no cell-autonomous effect of Rab27a knockdown (~50% decrease) on the synaptic integration of these cortical neurons. Nevertheless, we next examined whether Rab27a could have an effect in nearby non-electroporated neurons because Rab27a has been shown to control the release of intraluminal vesicles into the extracellular milieu, called small extracellular vesicles (sEVs) or exosomes, by regulating multivesicular body (MVB) docking and fusion with the plasma membrane [15,16,17,18,19,20,21]. There is accumulating evidence that sEVs have important roles during brain development, including the regulation of spine development, which was reported in cultured mouse and human neurons [22,23] (for reviews see References [24,25]). We thus examined the dendritic spine properties and synaptic activity of non-electroporated neurons. More specifically, we examined earlier born neurons that populate L4 and are thus in close contact with L2/3 electroporated neurons during perinatal development, but do not receive synaptic inputs from L2/3 neurons. Rab27a knockdown led to an increase in spine density and excitatory synaptic activity in non-electroporated L4 neurons that was accompanied by an increased number of activated neurons following whisker stimulation. This effect also led to the accumulation of MVBs in electroporated neurons in vivo, consistent with a decreased release of sEVs that we observed in vitro. Collectively, we identified a Rab27a-dependent release of sEVs that coordinates synapse development between distinct populations of cortical neurons, ultimately tuning responses to sensory stimulation.

## 2. Material and Methods

### 2.1. Animals

Research protocols were approved by the Yale University Institutional Animal Care and Use Committee. All experiments were performed on CD1 (Charles River) mice of either gender.

### 2.2. In Utero Electroporation (IUE) and Plasmids

Each DNA plasmid was diluted in sterile phosphate buffer saline (PBS) (pH 7.4) to a final concentration listed in Supplementary Table S1. The *Dcx*-Cre and pCAG-tdTomato plasmids were from References [26,27]. Timed pregnant mice at embryonic day (E) 13.5 or E15.5 were anesthetized with isoflurane. After exposing the uterine horns, ~1 μL of DNA solution, containing 0.01% fast green added as an injection tracer, was injected into the lateral ventricle via a pulled glass capillary. PBS-soaked tweezer-type electrodes (model 520, BTX) were then positioned on the head of the fetuses across the uterine wall and 6 square-pulses at 36 V (E13.5) or 42 V (E15.5), 50 ms duration, 950 ms intervals, were applied using a pulse generator (ECM830, BTX). Mice were prescreened for successful electroporation by detecting the expression of fluorescent protein markers on a fluorescence-enabled stereo microscope (SZX16, Olympus).

### 2.3. Electrophysiology

Slice physiology: Slices were obtained from P14 CD1 mice containing DsRed-positive neurons in layer (L) 4/5 and green fluorescent protein (GFP) expression in L2/3 cortical neurons generated by IUE at E13.5 and E15.5, respectively. On the day of the recording, mice were anesthetized with isoflurane and decapitated. Brains were quickly removed and immersed in ice-cold high-sucrose solution containing (in mM): 220 sucrose, 2.5 KCl, 6 MgCl_2_, 1 CaCl_2_, 1.23 NaH_2_PO_4_, 26 NaHCO_3_, and 10 glucose (gassed with 95% O_2_/5% CO_2_; 300 mOsm), or artificial cerebrospinal fluid (ACSF): 124 NaCl, 3 KCl, 1.25 NaH_2_PO_4_, 1 MgSO_4_, 26 NaHCO_3_, 10 dextrose, 2 CaCl_2_, 0.4 ascorbate, 4 Na-lactate, and 2 Na-pyruvate (290 ± 5 mOsm, pH 7.2). Coronal brain slices (300–350 µm-thick) were cut in ice-cold solution using a vibratome (Vibratome 1000) and incubated in ACSF at 32 °C for 45 min prior to returning to room temperature (20–25 °C). The recording solution was the ACSF solution detailed above or an ACSF solution containing (in mM): 124 NaCl, 2.5 KCl, 2 MgCl_2_, 2 CaCl_2_, 1.23 NaH_2_PO_4_, 26 NaHCO_3_, and 10 glucose (gassed with 95% O_2_/5% CO_2_; 300 mOsm). Experiments were performed at 30–33 °C using a dual-channel heat controller (Warner Instruments, Hamden, CT, USA). GFP- or tdTomato-expressing neurons were visualized with epifluorescence on an Olympus BX51WI microscope with a 40x water immersion objective (Olympus; LUMPlanFL/IR). Pipettes used for whole-cell recordings were pulled from thin-walled borosilicate glass capillary tubes (World Precision Instruments) using a P-97 micropipette puller (Sutter Instruments, Novato, CA, USA) and had resistances ranging from 4 to 7 MΩ when filled with a pipette solution containing (in mM) 145 K-gluconate, 1 MgCl_2_, 10 HEPES, 1.1 EGTA, 2 Mg-ATP, 0.5 Na_2_-GTP, and 5 Na_2_-phosphocreatine, or 4 KCl, 125 K-gluconate, 10 HEPES, 10 di-Tris-phosphocreatine, 1 EGTA, 0.2 CaCl_2_∙2H_2_O, 4 Na_2_-ATP, and 0.3 Na-GTP (pH 7.3 with KOH; 290–295 mOsm). Recordings were acquired with an amplifier (Axopatch 200B from Molecular devices or EPC-10 from HEKA Instruments), low-pass filtered at 5 kHz, 10x gained, and digitized at 20 kHz (Digidata 1320; Molecular Devices). Neurons, for which the series resistance was >20 MΩ and changed by >15%, were excluded from the analysis. Resting membrane potential was measured in current clamp mode without current injection within 5 s after break-in. Spontaneous synaptic currents were recorded in voltage-clamp mode for 5 min with cells clamped at −70 mV. Data were analyzed using Clampfit (Version 10; Molecular Devices) or PatchMaster 2.20 (HEKA Instruments).

Paired pulse recording in vitro: Neurons dissected from E15 cortices were cultured and nucleofected with ChR2-mCherry and *Rab27a* shRNA. ChR2-mCherry and empty shRNA vector (EV) were transfected as control. Paired pulse-evoked postsynaptic currents were recorded from non-fluorescent neurons receiving inputs from ChR2-mCherry-expressing neurons. Laser stimulation (470 nm) of 10 ms with an interval of 100 ms was used to activate presynaptic ChR2-expressed neurons and evoke postsynaptic currents on recorded neurons.

### 2.4. Spine Analyses

Spine analysis was performed blindly using the automated spine identifier in NeuronStudio [28] on confocal Z-stack images (0.2 µm-steps) acquired with a 60X oil Olympus Uplan SAPO (N.A. 1.35) objective on an Olympus Fluoview 1000 confocal microscope. Spine density and shape, including thin, stubby, and mushroom, were analyzed on two to three 100 μm-long segments of a secondary dendrite per neuron. Thin spines have a thin, long neck, and a small bulbous head, whereas mushroom spines have a larger head. Stubby spines are devoid of a neck and are prominent between postnatal development [29]. A mushroom type is defined as a spine with a head to neck ratio > 1.1 and a head diameter > 0.35 µm.

### 2.5. Novel, Enriched Environment for Whisker Stimulation

Littermate postnatal day (P) 21 mice that were electroporated at E15.5 with shRNA vectors were placed in a novel, enrichment environment for 2 h in the dark. After brain dissection, fixation, and slicing, coronal sections were immuno-stained for VGLUT2 and c-fos.

### 2.6. Immunostaining in Fixed Sections and Analysis

Brains were serially cut into 50 μm-thick coronal sections using a freezing microtome and stored in PBS + 0.01% sodium azide. For staining, free-floating sections were washed in PBS + 0.1% Triton X-100 (PBS-T) for 2 × 10 min and permeabilized in PBS + 0.3% Triton X-100 for 20 min. Sections were then incubated in blocking buffer (5% goat serum + 0.3% BSA + 0.3% Triton X-100 in PBS) for 1 h at room temperature, followed by incubation in primary antibodies (Supplementary Table S2) diluted in blocking buffer overnight at 4 °C. The following day, sections were washed in PBS-T three times for 10 min each and incubated in secondary antibodies (Supplementary Table S2) for 1 h at room temperature. Sections were then washed in PBS-T three times for 10 min each, incubated in DAPI for 10 min, rinsed in PBS, before being air-dried and cover-slipped with mounting media (ProLong Diamond Antifade Mounting medium; Invitrogen). Quantification of Rab27a and CD63 immunostainings were performed using two methods using NIH Image J software on Z-stack images obtained with a 60x (oil) objective on an Olympus Fluoview 1000 confocal microscope. One method measured the mean gray value of CD63 and Rab27a fluorescence in tdTomato-positive soma. A second method measured the number of pixels for each CD63 and Rab27a puncta above a set threshold and for tdTomato-positive soma to obtain surface area. Both methods gave similar results (data not shown). In addition, soma size was not affected by *Rab27a* shRNA and the fluorescence data were thus not divided by the measured soma size.

### 2.7. Neuronal Culture

Cerebral cortices from E15 mice (for nucleofection prior to culturing) were isolated after removing meninges and placed in ice-cold Hibernate E (Invitrogen, #A12476-01) supplemented with B27 (Invitrogen, #17504-044). The cortices were then incubated in pre-warmed and activated papain digestion solution (Worthington, #LK003150). After a 15 min digestion, neurons were dissociated and plated in MEM medium (Invitrogen, #11095-080), supplemented with 5% FBS (Invitrogen, #16000) and 0.6% glucose (Sigma, # G8769). Plating medium was replaced with neurobasal medium (Invitrogen, #21103-049) supplemented with B27 and GlutaMax-1 (Invitrogen, #35050-061) after neurons have attached. The medium was changed every 3 days.

### 2.8. Western Blot

Extracellular vesicles were lysed in reducing sample buffer (0.25 M Tris–HCl (pH 6.8), 40% glycerol, 8% SDS, 5% 2-mercaptoethanol, and 0.04% bromophenol blue) or non-reducing sample buffer (without 2-mercaptoethanol, for CD63), and then boiled for 5 min. Primary neurons were homogenized in RIPA buffer, 1x Halt Protease and phosphatase inhibitor cocktail (Thermo Fisher Scientific, Waltham, MA, USA), 5 mM EDTA, and 20 units/mL DNase I (Roche). All lysates were run on Tris-glycine gradient gels (Bio-Rad; #456-1084). Proteins were transferred to PVDF and blocked in 5% milk and incubated with primary antibodies in 0.25% BSA (concentrations of primary antibody are listed in Supplementary Table S2). HRP-conjugated anti-rabbit or anti-mouse were used as secondary antibodies. All density measurements were performed by using NIH Image J software.

### 2.9. Isolation of Small Extracellular Vesicles

We used sequential centrifugation procedures and filtration. Conditioned medium was first centrifuged at 300 g for 10 min at 4 °C to remove detached cells, followed by a 0.22 μm filtration, an ultracentrifugation at 120,000 g for 70 min at 4 °C using a fixed-angle bucket rotor (Beckman), and a washing step in PBS to eliminate contaminating proteins. Pellets were re-suspended in 50 µL PBS by syringing through a sterile 27-gauge needle to prevent clumping together. The isolated vesicular fractions were immediately characterized or stored at 80 °C for later use (no more than 1 week). The vesicle size distribution was accessed by nanoparticle tracking analysis with NanoSight NS300 (Malvern Panalytical Ltd., Malvern, UK).

### 2.10. Nucleofection of Primary Neurons

Mouse Neuron Nucleofector kit (Lonza, #VPG-1001) was used for nucleofection as previously described [30]. Briefly, following microdissection of the cortices and neuron dissociation, 4–6 × 10^6^ cells were spun down and re-suspended in nucleofection solution with 3–5 µg plasmids. DNA was transfected by nucleofector^®^ program 0–005. Neurons were then plated in MEM medium supplemented with 5% FBS and 0.6% glucose and maintained in neurobasal medium supplemented with B27 and GlutaMax-1.

### 2.11. Electron Microscopy

Electron microscopy (without additional staining) was performed by the imaging core facility in the department of Physiology at Yale. Purified vesicles were resuspended in 4% wt/vol paraformaldehyde in phosphate buffered solution (pH 7.4) and embedded for 20 min at room temperature in a formvar-carbon-coated grid. The embedded vesicles were washed in phosphate-buffered saline (PBS), fixed in 1% gluteraldehyde for 5 min, and stained with saturated aqueous uranyl oxalate. Samples were subsequently embedded in 0.4% wt/vol uranyl acetate and 1.8% wt/vol methylcellulose on ice for 10 min. Samples were dried at room temperature prior to visualization with a Carl Zeiss 910 electron microscope (Carl Zeiss Microscopy, Thornwood, NY, USA).

### 2.12. Statistical Analyses

Data were plotted in Prism 7 (GraphPad Software, Inc., San Diego, CA, USA). Statistical significance was determined using the unpaired two-tailed Student’s t-test or one-way analysis of variance (ANOVA) with Tukey’s post-hoc test with *p* < 0.05 for significance for all experiments. Data are presented as mean ± SEM including the data points in some of the plots. Each experiment was reproduced at least three times, e.g., three mice or three sets of culture. The Student t-tests were all two-sided and unpaired. Normality tests were performed using the D’Agostino-Pearson omnibus test.

## 3. Results

### 3.1. Rab27a Is Present in Developing Cortical Neurons, but Decreasing Rab27a Expression has no Cell-Autonomous Effect on the Synaptic Integration of L2/3 Pyramidal Neurons

**Figure 1:**
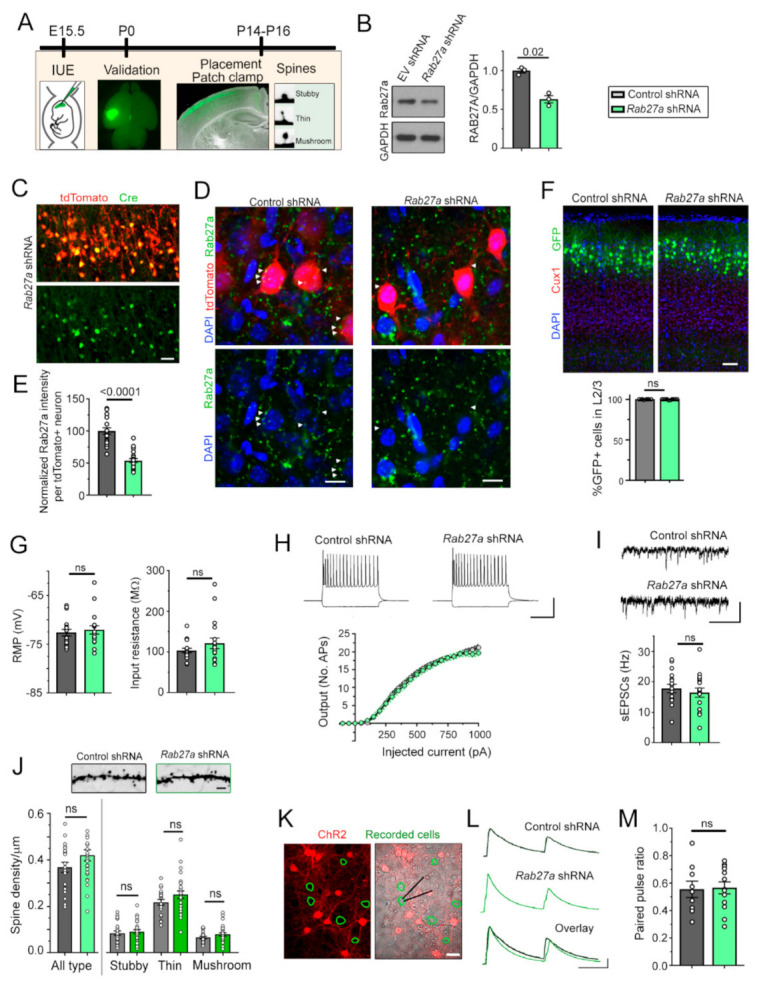
Rab27a knockdown does not affect cortical neuron placement and excitatory synaptic integration in a cellautonomous manner. (**A**) Diagram of the experimental paradigm. IUE: in utero electroporation. (**B**) Immunoblots of Rab27a, GAPDH from neurons containing empty vector (EV, control) or *Rab27a* shRNA and quantification of the blots shown in (**A**). ttest, N = 3 sets of cultures. (**C**) Images of tdTomato fluorescence and Cre immunostaining in coronal section from a (postnatal day) P14 mouse electroporated at embryonic day (**E**) 15 with *Dcx*-Cre, tdTomato, and *Rab27* shRNA. (**D**,**E**) Z-stack images of tdTomato fluorescence, DAPI, and Rab27a immunostaining in coronal sections from P14 mice electroporated with either control or Rab27a shRNA (**D**). Plots of the normalized Rab27a intensity per cell in both conditions (**E**). (**F**) Top: images of Cux1 immunostaining and GFP^+^ neurons containing control or *Rab27a* shRNA in coronal sections from P14 mice. Blue: DAPI overlay. Bottom: graph of the % of GFP^+^ neurons expressing Cux1 and in layer (L) 2/3, the placement of neurons electroporated at E15.5. (**G**) Bar graphs of the mean resting membrane potentials (RMP) and input resistance of neurons expressing control or *Rab27a* shRNA. (**H**) Representative current-induced firing of recorded neurons and resulting input–output curves in both conditions. (**I**) Representative recordings of sEPSCs in neurons containing control or *Rab27a* shRNA and bar graphs of sEPSC frequency in both conditions. (**J**) Images of spines in each condition, and density of spines (all types) and of each individual type of spines in GFP^+^ neurons containing control (n = 21) or *Rab27a* (n = 26) shRNA. (**K**) Images of tdTomato^+^ neurons co-expressing ChR2 with control or *Rab27a* shRNA. The green circles delineate recorded neurons. Cortical neurons were nucleofected with *Rab27a* or control shRNA, and a plasmid encoding channelrhodopsin (ChR2) fused to mCherry. After plating, non-mCherry neurons were recorded at DIV 14 while light stimulation triggered spiking of ChR2-exressing neurons, resulting in synaptic release. (**L**) Representative examples of paired pulse traces induced by light stimulation of ChR2 in tdTomato^+^ neurons expressing control or *Rab27a* shRNA that are neighboring recorded neurons. (**M**) Plots of the mean paired pulse ratio. n = 9 neurons in control condition and n = 13 neurons in the *Rab27a* shRNA condition. Unpaired t-test and one-way ANOVA, ns: not significant and ** *p* < 0.01, *** *p* < 0.001, **** *p* < 0.0001. Scale bars: 200 µm (**C**), 30 µm (**D**), 100 µm (**F**), 50 mV, 200 ms (**H**), 20 pA, 200 ms (**I**), 2.5 µm (**J**), 25 µm (**K**), and 50 pA, 100 ms (**L**). Plots are mean ± SEM including data points.

To examine the function of Rab27a on the development of cortical neurons, we decreased Rab27a levels using a knockdown strategy in vivo with in utero electroporation (IUE, Figure 1A). We first tested the knockdown efficiency of shRNA vectors (conditional pSico [31] co-expressed with pCAG-Cre) against Rab27a in cultured cortical neurons. Three days following nucleofection of cultured neurons, *Rab27a* shRNA significantly decreased Rab27a levels compared to a control empty vector (Figure 1B). In addition, we found no compensatory increase in Rab27b in the *Rab27a* shRNA condition (Supplementary Figure S1). We then used IUE to express conditional *Rab27a* shRNA plasmid or an empty vector control in radial glia at embryonic day (E) 15–15.5, targeting late-born pyramidal neurons that populate layer (L) 2/3 in the somatosensory cortex. In both conditions, we co-electroporated an inducible GFP plasmid and a vector encoding Cre recombinase (Cre) under the neuronal promoter doublecortin (*Dcx*) to induce recombination selectively in developing neurons, but not in radial glia [26]. Immunostaining for Cre showed that nearly all electroporated cells displayed Cre expression (Figure 1C). We also immuno-stained for Rab27a in both control and *Rab27a* shRNA conditions (Figure 1D). Analysis of Rab27a fluorescence per cell or Rab27a puncta size revealed a significant decrease (47%) of Rab27a expression in the *Rab27a* shRNA condition (Figure 1E). Decreased Rab27a expression did not alter the placement of L2/3 pyramidal neurons (Figure 1F). Whole-cell patch-clamp recordings of electroporated neurons revealed that *Rab27a* shRNA did not alter their resting membrane potential, input resistance, and intrinsic excitability (Figure 1G,H). In addition, the frequency of spontaneous excitatory postsynaptic currents (sEPSCs) was not altered by *Rab27a* knockdown (Figure 1I). Since dendritic spines are the unitary sites of excitatory synaptic inputs, we examined their density. Consistent with the sEPSC data, knocking down Rab27a did not alter the density and shape distribution of spines in electroporated neurons (Figure 1J). Considering that Rab27 regulates synaptic release in C. elegans and squid [10,11], we examined whether *Rab27a* shRNA would affect synaptic vesicular release from cortical neurons in vitro. We co-electroporated channelrhodopsin (ChR2) with the shRNA and induced action potentials followed by vesicular release from neurons expressing *Rab27a* shRNA. Using patch-clamp recordings of neighboring non-electroporated neurons in the same cultures (Figure 1K), we found that *Rab27a* shRNA did not affect paired pulse ratio, a read-out of release probability (Figure 1L,M). Collectively, these data suggest that decreasing Rab27a levels in cortical neurons did not affect their migration, spine development, and synaptic connectivity in a cell-autonomous manner.

### 3.2. Decreasing Rab27a Levels in L2/3 Neurons Increases L4 Neurons’ Excitatory Synaptic Inputs and Activation by Whisker Stimulation

**Figure 2:**
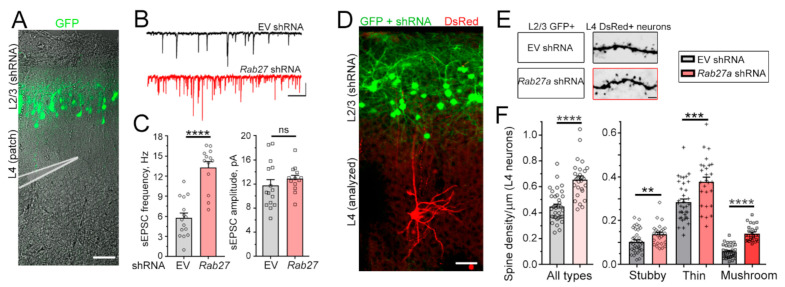
Rab27a knockdown in L2/3 (late-born) neurons alters glutamatergic transmission in L4 (earlier born) pyramidal neurons. (**A**) Confocal images illustrating GFP+ L2/3 neurons (IUE at E15.5 with GFP) and diagram of a patch pipette over L4. (**B**) Representative whole-cell patch-clamp recordings at −70 mV of L4 pyramidal neurons in P14 acute slices containing GFP^+^ L2/3 neurons expressing empty vector (EV, control) or *Rab27a* shRNA. (**C**) Bar graphs of the sEPSC frequencies (n = 15–21 L4 neurons for each condition). Unpaired t-test. (**D**) Confocal images illustrating GFP^+^ L2/3 neurons (IUE at E15.5 with GFP) and DsRed^+^ L4 neurons (IUE at E13.5 with an inducible DsRed vector (pCALNL-DsRed) and low concentration of Dcx-Cre). (**E** and **F**) Images of spines (**E**) and quantification (**F**) of spine density (all types) and each type in DsRed^+^ neurons in P14 slices containing GFP^+^ neurons expressing either control (n = 36 neurons) or *Rab27a* (n = 29) shRNA (4 mice per condition). Unpaired Student’s t-test and one-way ANOVA with Tukey’s post-hoc test. Unpaired t-test. Plots are mean ± SEM. ****: *p* < 0.0001, ***: *p* < 0.001, **: *p* < 0.01, and ns: not significant. Scale bars: 100 µm (**A**), 20 pA, 250 ms (**B**), and 2 µm (**E**).

Considering Rab27a’s role in vesicular trafficking, including the fusion of MVB resulting in the release of intraluminal vesicles (ILs) into the extracellular space that are then called sEV or exosomes [15,16,17,18,19,20,21], we investigated whether synaptic and biophysical properties of non-electroporated neurons nearby *Rab27a* shRNA-expressing neurons were altered. We examined L4 neurons because these neurons are born earlier than L2/3 neurons and as such, late-born neurons destined for L2/3 neurons migrate through deep cortical layers containing L4 neurons to reach their final location. In addition, the dendritic trees of L2/3 and L4 pyramidal neurons overlap during the neonatal period, and their axons travelled through each other’s dendritic tree and soma territories [32]. In addition, L4 neurons do not receive synaptic inputs from L2/3 neurons [33]. Patch-clamp recordings were obtained at P14 to examine potential changes in neuronal biophysical properties and synaptic inputs (Figure 2A). At this developmental age (P14), cortical pyramidal neurons are still acquiring dendritic spines (Supplementary Figure S2). Decreasing Rab27a levels in L2/3 neurons had no effect on the resting membrane potential, membrane resistance, and intrinsic excitability of L4 neurons (data not shown). However, it led to a significant increase in the frequency of sEPSCs in nearby L4 neurons without affecting their amplitude (Figure 2B,C). To evaluate spine properties, we used a dual IUE approach to conditionally express *Rab27a* shRNA in L2/3 neurons (E15.5 IUE) and DsRed in L4 neurons in the same animal using an inducible vector and *Dcx*-Cre (E13.5 IUE, Figure 2D). Expressing *Rab27a* shRNA in L2/3 neurons significantly increased spine density in L4 neurons, including the density of mushroom spines, which are considered mature and stable spines (Figure 2E,F).

**Figure 3:**
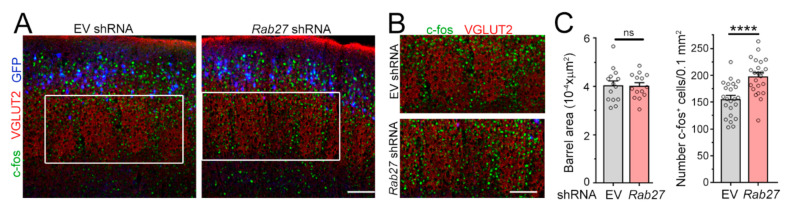
Decreasing Rab27a in L2/3 neurons leads to an increase in the number of L4 neurons activated by Whisker stimulation. (**A**) Images of c-fos (green), vesicular glutamate transporter 2 (VGLUT2) (red), and GFP (pseudo-colored blue) in slices from mice electroporated with EV or *Rab27a* shRNA in L2/3 neurons. (**B**) Zoom of c-fos and VGLUT2 images in the white rectangle in (**A**). (**C**) Bar graph of the cross-sectional area of barrels and the density of c-fos^+^ cells under both conditions (n = 4 mice with EV and n = 5 mice with *Rab27a* shRNA, n = 23 slices in each condition). The barrels were delineated using VGLUT2 immunostaining. Unpaired t-test. Plots are mean ± SEM including data points. ****: *p* < 0.0001 and ns: not significant. Scale bars: 100 µm (**A**) and 80 µm (**B**).

We next speculated that an increased number of excitatory synaptic inputs onto L4 neurons would lead to enhanced sensory responses upon whisker stimulation. L4 neurons in the somatosensory barrel cortex receive sensory information transmitted from the whiskers to the thalamus and provide a well-defined system to measure cellular responses to sensory inputs [34]. To test this, we placed P21 mice in an enriched environment to increase whisker stimulation and examined the number of L4 neurons immuno-positive for the immediate early gene c-Fos, a well-established read-out of neuronal activity [35] (Figure 3A,B). We found that reducing Rab27a expression in L2/3 neurons did not affect the anatomy of the barrels, but it led to a significant increase in the number of c-Fos-positive L4 neurons in the barrel cortex following whisker stimulation (Figure 3C). Collectively, these data indicate that decreasing Rab27a levels had a non-cell autonomous effect on surrounding cortical neurons.

### 3.3. Decreasing Rab27a Levels in Cortical Neurons Reduces the Release of sEVs

**Figure 4:**
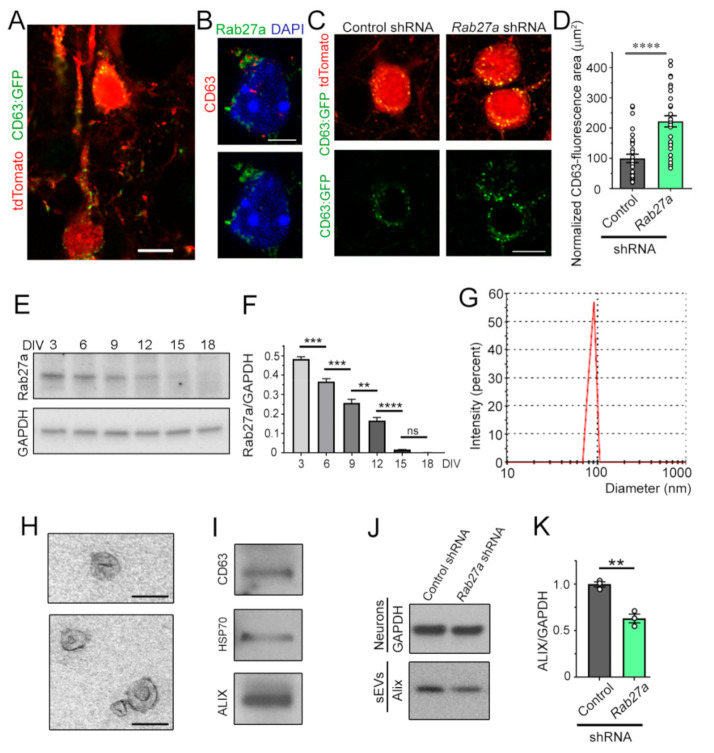
Rab27a regulates the release of small extracellular vesicles (sEVs) from developing cortical neurons. (**A**) Image of CD63:GFP and tdTomato fluorescence in late-born pyramidal neurons at P7 following IUE at E15. (**B**) Co-immunostaining for CD63 (red) and Rab27a (green) in a pyramidal neuron (P7 coronal section). (**C**) tdTomato and CD63:GFP fluorescence in pyramidal neurons from P7 mice that received CD63:GFP and either empty vector (control) or *Rab27a* shRNA following IUE at E15.5. (**D**) Plot of the normalized area occupied by CD63 immunostaining. (**E**,**F**) Immunoblots for Rab27a and GAPDH (**E**) from primary cortical neurons at different DIV and quantification of the blots (**F**). (**G**) Histogram of size distribution of sEVs determined by Nanoparticle Tracking Analysis. (**H**) Electron micrographs of purified sEVs. (**I**) Immunoblots for markers of sEVs. (**J**,**K**) Immunoblots of Alix from sEVs and GAPDH from neurons containing control (empty) shRNA vector or *Rab27a* shRNA (**J**), and quantification of the blots (**K**). These data were from the same cell cultures performed for Figure 1B. Plots are mean ± SEM. **: *p* < 0.01, ***: *p* < 0.001, ****: *p* < 0.0001, and ns: not significant, Student’s t-test. Scale bars: 15 µm (**A**,**C**), 7 µm (**B**), and 125 µm (**H**).

Considering the non-cell-autonomous effects of Rab27a knockdown, we examined whether Rab27a controls the release of sEVs from neurons, as shown in non-neuronal cells [15,16,17,18,19,20,21]. To visualize MVBs in vivo, we used IUE at E15.5 to express tdTomato and a vector encoding CD63 fused to GFP. CD63 is expressed in MVB and intraluminal vesicles (ILs). At P7, CD63:GFP fluorescence was detected as puncta inside the soma and dendrites of tdTomato-positive pyramidal neurons, as expected, based on the known localization of MVBs [36] (Figure 4A). Immunostaining for Rab27a and CD63 revealed that Rab27a expression was associated with, and surrounded, CD63-positive puncta in P7 cortical neurons (Figure 4B). These data are consistent with Rab27a being involved in the fusion of MVBs with the plasma membrane, but not being contained inside MVBs or ILs [37]. Importantly, Rab27a knockdown led to an increase in the number of CD63-positive vesicles (Figure 4C,D), suggesting the accumulation and decreased release of IL/sEV. To further confirm this interpretation, we performed in vitro experiments. First, we examined at which time points in cultured cortical neurons (from days in vitro (DIV) 3 to 18) Rab27a levels were the highest. Using immunoblotting, Rab27a expression was the highest at DIV 3 and progressively decreased as neurons matured, consistent with the low expression in adult brains (Figure 4E,F). Next, we examined the effect of Rab27a knockdown on the release of sEVs at DIV 3. To isolate sEVs, the extracellular medium of DIV 3 cortical neurons was collected and subjected to filtration and sequential centrifugations [37,38]. The isolated vesicles in the sEV fraction contained nano-sized sEVs (also known as exosomes) based on their mean diameter (range: 60–115 nm, Figure 4G, smaller than the diameter of shedding microvesicles and apoptotic bodies [18,19,37,39,40,41,42]), their cup-like morphology assessed by electron microscopy (Figure 4H), and the presence of markers of MVB-generated sEVs, e.g., CD63 and Alix (Figure 4I) [37,43]. Nucleofection of *Rab27a* shRNA in primary cortical neurons reduced the amount of released Alix (normalized to GAPDH from cultured neurons), suggesting that reducing Rab27a levels decreases the quantity of released sEVs from cortical neurons (Figure 4J,K).

## 4. Discussion

Rab27a is one of two Rab27 small GTPases that are involved in intracellular vesicle trafficking. The role of Rab27a in the mammalian brain has not been examined, despite previous work showing a critical role of Rab27a in the development of synapses in invertebrates and the association of *RAB27A* copy number variations with simplex autism [9]. The lack of studies on Rab27a may stem from the fact that it was thought that Rab27a expression was very low in the adult brain [44,45]. Here, we confirmed that Rab27a levels are low in mature neurons compared to developing neurons in vitro. In particular, Rab27a levels progressively decreased as neurons acquired spines and synapses in vitro, suggesting that Rab27a expression may regulate spine development in a cell-autonomous manner. However, knocking down Rab27a in developing cortical neurons did not affect their spine and synapse development, suggesting that Rab27a does not regulate spine development in a cell-autonomous manner. However, Rab27a expression was decreased by about 50%, which may not be sufficient to affect neuronal development. Rab27b has been shown to compensate for the decrease in Rab27a in melanocytes of Rab27a knockout mice [45]. Here, we found no compensatory increase in Rab27b expression in cultured neurons expressing *Rab27a* shRNA. Collectively, decreasing Rab27a expression in cortical neurons had no net effect on the synaptic integration of *Rab27a* shRNA-expressing cortical neurons in vivo. In light of the known role of Rab27a in regulating sEV release, we next examined whether Rab27a knockdown would have a non-cell autonomous effect. Decreasing Rab27a levels in late-born L2/3 neurons led to a significant increase in excitatory synaptic activity as well as the density of dendritic spines in earlier born L4 neurons. Collectively, these data suggest that Rab27a controls a form of paracrine signal that limits spine development and excitatory synaptic inputs across neuronal populations.

To examine one potential mechanism of this non-cell-autonomous effect, we examined and validated that Rab27a regulates sEV release. Rab27a-positive puncta in P7 cortical sections were in close proximity to CD63-positive puncta that labeled MVB, which have been shown to release sEVs. Knocking down Rab27a led to the accumulation of CD63-positive puncta in cortical neurons, as previously reported in HeLa-CIITA cells [15]. Although using a plasmid encoding CD63, which increases CD63 levels, would be an issue if studying the function of endogenous CD63, this is not the case here since we compare CD63:GFP expression under two conditions. In addition, considering that CD63:GFP and tdTomato expression are under the same CAG promoter and *Rab27a* shRNA had no effect on tdTomato expression, we do not expect an artifactual effect of *Rab27a* shRNA on the plasmid-driven expression of CD63:GFP. These data suggest that Rab27a knockdown results in a decrease in the release of sEVs. To further ascertain the in vivo data, we used an in vitro system and found that knocking down Rab27a in DIV 3 cortical neurons led to a significant decrease in the release of Alix-positive sEVs. These data suggest that Rab27a regulates intracellular CD63-positive vesicle trafficking and the release of sEVs in developing cortical neurons. This effect may contribute to the in vivo non-cell-autonomous effect of *Rab27a* shRNA on L4 neurons. Indeed, sEVs package molecules, including protein and microRNA that could influence spine and synapse development of neighboring neurons [24,40,46]. For example, in the Drosophila larval neuromuscular junction, exosome-mediated transport of Wnt signals coordinates the development of pre- and post-synaptic components [47,48]. A recent study also reported that treatment with sEVs (exosomes) isolated from human-induced pluripotent stem cell (hiPSC)-derived neurons increase synapse density in hiPSC-derived neurospheres [23]. The molecules involved in this regulation were not examined, but the authors performed a proteomic screen that identified more than 2500 proteins in sEVs, providing potential candidates involved in spine regulation. Another recent study identified the presence of the epigenetic regulator HDAC2 in sEVs that regulated spine development in cultured cortical neurons [49]. Identifying the mechanisms of spine regulation by sEV (that include exosomes) in our system is outside the scope of the present study. Collectively, our findings show that Rab27a, possibly via regulating sEV release, controls a novel form of paracrine communication that precedes and regulates the establishment of synaptic transmission.

## Supplementary Materials

The following are available online at https://www.mdpi.com/2073-4409/10/3/622/s1, Figure S1: Rab27a shRNA does not alter Rab27b levels in culture cortical neurons, Figure S2: Spine devel-opment is undergoing at P14, Table S1: List of plasmids, Table S2: List of antibodies.
