# Supplementary material for: Rab27a-Dependent Paracrine Communication Controls Dendritic Spine Formation and Sensory Responses in the Barrel Cortex
Source: Cells. 2021 Mar 11;10(3):622. doi: 10.3390/cells10030622 (PMC8000154; doi:10.3390/cells10030622)
Supplement: Supplementary file 1 [file cells-10-00622-s001.pdf]

Supplemental Material

Table S1. List of plasmids

Plasmids	Sequences or sources
shRNA	
pSico-*Rab27a*	GCTGCCAATGGGACAAACATA
Expression vector	
DCX-Cre-IRES-GFP	Lin et al., 2016
pCAG-GFP	https://www.addgene.org/11150/
pCAGGS-ChR2	https://www.addgene.org/15753
pCAG-tdTomato	https://www.addgene.org/83029/ (Pathania et al., 2012)
pCALNL-DsRed	https://www.addgene.org/13769/
pCALNL-GFP	https://www.addgene.org/13770/
pSico	https://www.addgene.org/11578/

Table S2. List of antibodies

Antibody	Company	Catalog Number	Dilution
Primary antibody			
Alix	Cell Signaling Technology	2171	1:3,000
Rab27a	Cell Signaling Technology	69295	1:1000
	Abcam	ab55667	1:200 (IF)
CD63	Abcam	ab217345	1:200 (IF, in vitro)
	Santa Cruz Biotechnology	sc-5275 and sc-15363	1:200 (IF, in vivo) and 1:500
c-fos	Cell Signaling Technology	2250	1:1,000
Cre	Cell Signaling Technology	15036	1:1,000 (IF)
Cux1	Santa Cruz Biotechnology	sc-13024	1:100 (IF)
GAPDH	Santa Cruz Biotechnology	sc-25778	1:5,000
GFP	Cell Signaling Technology	2956	1:5,000
VGLUT2	Synaptic Systems	135421	1:1,000
Secondary antibody			
anti rabbit	Cell Signaling Technology	7074	1:5,000
anti mouse	Cell Signaling Technology	7076	1:5,000
488 anti chicken	ThermoFisher	A-11039	1:1,000 (IF)
633 anti rabbit	ThermoFisher	A-21070	1:1,000 (IF)

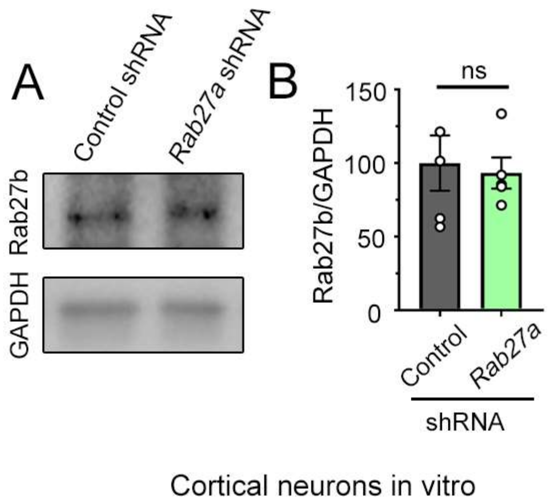

Figure S1: *Rab27a* shRNA does not alter Rab27b levels in culture cortical neurons. (A) Immunoblots of Rab27b, GAPDH from neurons containing empty vector (control) or *Rab27a* shRNA. **(B)** Quantification of the blots shown in (A). t-test, N=3 sets of cultures.

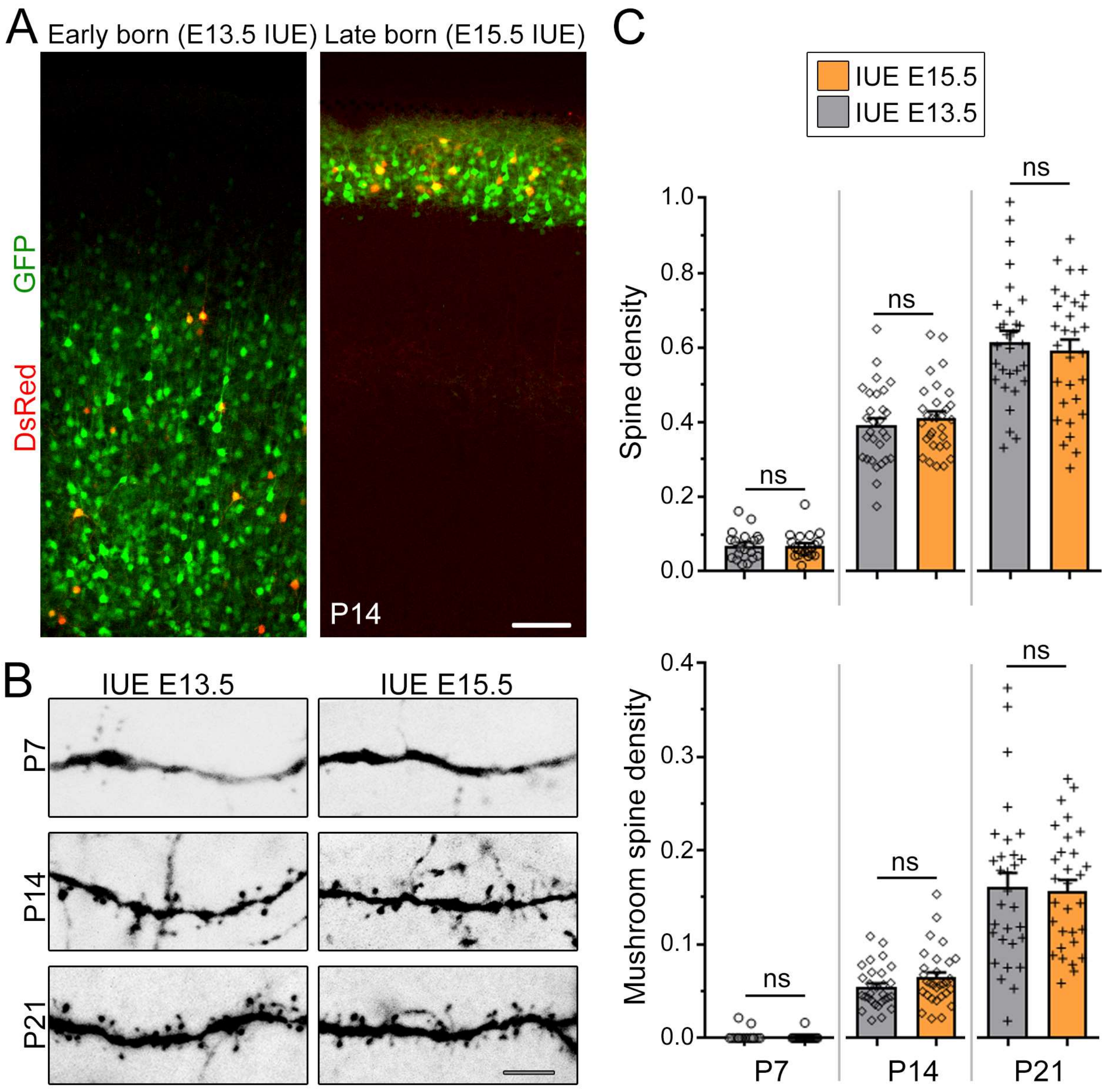

Figure S2: Spine development is undergoing at P14. (A) Confocal images of GFP and DsRed fluorescence in coronal sections from P14 mice electroporated at either E13.5 to target L4/5 neurons or E15.5 to target L2/3 neurons. Embryos were electroporated with pCAG-GFP, pCALNL-DsRed (a LoxP-based inducible vector), and *Dcx*-Cre plasmid at low concentration to induce DsRed expression in only a few neurons per section. **(B)** Confocal images of dendritic sections in L4/5 and L2/3 neurons containing filopodia or spines at three different postnatal ages indicated on the left. **(C)** Bar graphs (with each data point) of the density of spines in L4/5 (grey) and L2/3 (orange) neurons at the three developmental postnatal ages examined (per layer set: n=20

neurons at P7, n=29 at P14 and n=29 at P21, from 4 mice). Unpaired 2-tail t-test, ns: not significant. Scale bars: 200 µm (A) and 5 µm in (B). Plots are mean ± SEM.
